# Detection of Chronic Wasting Disease Prions in Fetal Tissues of Free-Ranging White-Tailed Deer

**DOI:** 10.3390/v13122430

**Published:** 2021-12-03

**Authors:** Amy V. Nalls, Erin E. McNulty, Amber Mayfield, James M. Crum, Michael K. Keel, Edward A. Hoover, Mark G. Ruder, Candace K. Mathiason

**Affiliations:** 1 Department of Microbiology, Immunology and Pathology, College of Veterinary Medicine and Biomedical Sciences, Colorado State University, Fort Collins, CO 80523, USA; amy.nalls@colostate.edu (A.V.N.); erin.mcnulty@colostate.edu (E.E.M.); amber.mayfield@outlook.com (A.M.); edward.hoover@colostate.edu (E.A.H.); 2 Wildlife Resources Section, West Virginia Division of Natural Resources, Elkins, WV 26241, USA; James.M.Crum@wv.gov; 3 Department of Pathology, Microbiology and Immunology, School of Veterinary Medicine, University of California, Davis, CA 95616, USA; mkkeel@ucdavis.edu; 4 Southeastern Cooperative Wildlife Disease Study, College of Veterinary Medicine, University of Georgia, Athens, GA 30602, USA; mgruder@uga.edu

**Keywords:** prions, chronic wasting disease, mother-to-offspring transmission, sPMCA, RT-QuIC, fetal tissues

## Abstract

The transmission of chronic wasting disease (CWD) has largely been attributed to contact with infectious prions shed in excretions (saliva, urine, feces, blood) by direct animal-to-animal exposure or indirect contact with the environment. Less-well studied has been the role that mother-to-offspring transmission may play in the facile transmission of CWD, and whether mother-to-offspring transmission before birth may contribute to the extensive spread of CWD. We thereby focused on a population of free-ranging white-tailed deer from West Virginia, USA, in which CWD has been detected. Fetal tissues, ranging from 113 to 158 days of gestation, were harvested from the uteri of CWD+ dams in the asymptomatic phase of infection. Using serial protein misfolding amplification (sPMCA), we detected evidence of prion seeds in 7 of 14 fetuses (50%) from 7 of 9 pregnancies (78%), with the earliest detection at 113 gestational days. This is the first report of CWD detection in free ranging white-tailed deer fetal tissues. Further investigation within cervid populations across North America will help define the role and impact of mother-to-offspring vertical transmission of CWD.

## 1. Introduction

Investigations into the transmission dynamics of chronic wasting disease (CWD) have primarily focused on the presence of the infectious agent (prions) in bodily fluids and excretions of infected cervids [1,2,3,4]. The prevailing hypothesis is that contact with prions shed by CWD-infected cervids via animal-to-animal contact, or presumed ingestion of prions from contaminated environments represent the majority of CWD spread among cervids. Less well-studied has been the role of prion transmission from mother to offspring.

Prion transmission from mother to offspring has been demonstrated for sheep scrapie [5,6,7]. The first evidence for maternal prion transmission came from observational studies revealing that the incidence of scrapie infections increased during the lambing season [6]. Further investigation led to reports of scrapie-associated aberrant prion protein (PrP^Scrapie^) deposition within maternal and fetal tissues [8,9,10,11,12,13,14,15], as well as the presence of prion infectivity within placental tissues [16,17], fetal tissues [18], embryos [14], and milk of scrapie-infected dams [19,20,21,22,23]. This effectively demonstrated a role for maternal transmission, with increasing evidence for prebirth fetal exposure to scrapie.

The evidence for prebirth CWD exposure and transmission is also mounting. We previously reported mother-to-offspring transmission, and the detection of CWD prion seeding activity in maternal reproductive and in utero-derived fetal tissues harvested from experimentally-infected Reeves’ muntjac (*Muntiacus reevesi*) [24]. Further studies in our maternal infection model led to the discovery of the infectious agent within the pregnancy microenvironment (uterus, placentomes, amniotic fluid) [25]. This piqued our interest to investigate the biological relevance of mother-to-offspring transmission in free-range cervid populations. Collaborations with the U.S. National Park Service led to the demonstration of prion seeding activity within in utero-derived fetal tissues harvested from naturally exposed free-ranging CWD+ asymptomatic Rocky Mountain elk (*Cervus canadensis*) cows [26].

To explore the potential differences between cervid species in prebirth dissemination of prions from mother-to-offspring we extended our studies to examine in utero-derived fetal tissues from white-tailed deer (*Odocoileus virginianus*). Here we used serial protein misfolding cyclic amplification (sPMCA) to determine if prion seeding activity is present in fetal tissues harvested from asymptomatic CWD+ does from West Virginia, USA. We report, for the first time, prion seeding activity within fetal tissues of naturally exposed free-ranging asymptomatic CWD+ white-tailed deer.

## 2. Materials and Methods

### 2.1. White-Tailed Deer Tissues: Origin, Handling, and Fetal Prnp Genotyping

Samples used in this project were opportunistically collected from naturally-infected, asymptomatic, CWD-positive white-tailed deer in Hampshire County, West Virginia. The deer were collected in March and April of 2011 and 2012 by West Virginia Division of Natural Resources (WVDNR) during targeted surveillance and management activities. Doe CWD status was confirmed by immunohistochemistry (IHC) on medial retropharyngeal lymph node (RPLN) and obex according to standard protocols at the Southeastern Cooperative Wildlife Disease Study (SCWDS; University of Georgia, Athens, GA, USA). Fourteen whole white-tailed deer fetuses were collected from nine CWD-positive does, including five sets of twins and four singles. Control fetal tissues (four singles) obtained from SCWDS, were collected from four CWD-negative does harvested in Georgia, USA, a region where CWD is not known to occur, as part of disease surveillance efforts. All does included in this study were collected by a sharpshooting team (rifle shots to the neck of the deer) and taken to a facility necropsy room between 9 and 14 h after collection. Great care was taken to mitigate cross contamination between does by using deer specific gloves, single use blades, scissors, and forceps. A team of three people were at the necropsy. All personnel wore PPE including gloves, Tyvek suits, and booties. One individual extracted tissues from the head of the deer, a second individual collected tissues from the body cavity of the deer, and a third person bagged and labeled the tissues. The fetuses were removed from the uterus by one person wearing clean gloves while the uterus was still in the body cavity. Each fetus (Table 1) was measured and placed in a large ziplock bag held open by the third person, which was labeled and immediately placed in a 0 °F freezer. Once the CWD status of the does was confirmed, frozen fetuses were shipped to Colorado State University (CSU). After thawing, tissues from each fetus were harvested using single-use, animal- and tissue-specific blades and forceps to prevent cross-contamination, as previously described [24]. The following tissues were analyzed from each fetus: brain, ileum, popliteal lymph node, thymus, and liver. Tissue homogenates were made at 2.5–20% *w/v*, depending on tissue sample size (Table 2), in cold, sterile 0.1 M PBS containing 0.1% Triton-X, using 0.5 mm Zirconium Oxide beads in a BBX24B Bullet Blender Blue Homogenizer (Next Advance, New York, NY, USA). All samples were coded, double-blinded, and subjected to sPMCA as previously described [26]. Sample identities were not revealed until after all analyses were completed. *Prnp* genotype at codon 96 was determined for the fetuses as previously described [27].

### 2.2. Gestational Age

The fetal gestational age was determined by use of the Hamilton equation; Age (days) = (body length [in mm] × 0.32) + 36.82 [28].

### 2.3. Serial Protein Misfolding Cyclic Amplification (sPMCA)

sPMCA was performed using one of two methods, as the assay has evolved over time in our laboratory. For both methods, a 10% normal brain homogenate (NBH) in 0.1 M PBS buffer (pH 7.5, with 1% Triton X-100) was prepared from whole brains collected from clinically healthy naïve transgenic mice (<4 months of age) that overexpress cervid PrP (TgCerPrP-E2265037) to serve as a substrate for the PrP^C^ to PrP^CWD^ seeded conversion reaction (prion seeding activity) in sPMCA as previously described [29]. Fetal tissue homogenate (30 μL; ranging from 2.5 to 20% *w/v*; Table 2) was added to 50 μL 10% *w/v* NBH and subjected to either sPMCA method 1 (run in 2013): seven 24 h-rounds of sonication with each round equaling 288 cycles of 10 s sonication/5 min incubation, or sPMCA method 2 (run in 2021): one round of 72 h (144 cycles) followed by four rounds of 24 h (48 cycles each) of 30 s sonication/29 min 30 s incubation. Both sPMCA methods were performed at 37 °C with Misonix sonicator setups. After each round, 30 μL amplified material was transferred to 0.2 mL PCR tubes containing 50 μL 10% NBH, two 2.38 mm and three 3.15 mm Teflon beads (McMaster-Carr, Elmhurst, IL, USA). sPMCA method 1 reactions were assessed by western blotting, whereas sPMCA method 2 reactions were assessed by RT-QuIC.

### 2.4. Western Blotting

The seventh round of sPMCA method 1 reaction was assessed by western blot for the detection of the aberrant prion protein (PrP^CWD^) as previously described [24,26]. These samples were run alongside known experimentally inoculated cervid CWD positive and negative laboratory control brain homogenates, both unamplified (4 μL loaded) and sPMCA method 1 amplified (8 μL loaded). Samples were mixed with proteinase K (Invitrogen, Waltham, MA, USA) at 20 μg/mL final concentration, and incubated at 37 °C for 30 min, followed by an additional 10 min at 45 °C with shaking. Samples were mixed with Reducing Agent (10X)/LDS Sample Buffer (4X) (Invitrogen, Waltham, MA, USA) per manufacturer’s instructions, heated to 95 °C for 5 min, then run through a 12% Bis-Tris gel at 100 volts for 2 h. Proteins were transferred to a polyvinylidene fluoride (PVDF) in a Trans-Blot Turbo Transfer System (BioRad, Hercules, CA, USA). The membrane was blocked with Casein TBS Blocking buffer (Thermo Scientific, Waltham, MA, USA) and probed with BAR-224-HRP antibody (0.2 µg/mL) as described above, then developed with ECL Plus Western Blotting Detection Reagents (GE) and viewed with the ImageQuant LAS-4000 (GE).

### 2.5. Real Time Quaking Induced Conversion (RT-QuIC)

RT-QuIC was performed as previously described [29], using truncated Syrian hamster recombinant protein encoding residues 90–231, as a readout of sPMCA method 2 for the detection of prion seeding activity. Briefly, sPMCA 5th round product was diluted 1:1000 in 0.1% SDS/PBS. Two microliters of each diluted sample (including amplified negative and positive laboratory and fetal matched tissue negative controls) were run in triplicate or quadruplicate on 2–3 plates by two investigators at 42 °C for 62 h. The threshold was set at 5 SD above the mean of the initial 5 readings. The inverse of the time when the reaction reached the threshold (1/time to threshold) was then used to determine the amyloid formation rate. Statistical analyses were run in Prism v9, GraphPad Software, La Jolla, CA, USA. A Mann-Whitney test was used to generate *p*-values (those <0.05 were considered significant) by comparing the median of the fetal tissue sample rates to the median of tissue-matched negative control rates.

## 3. Results

### 3.1. Gestational Aging

Using the Hamilton equation [28], the fetal age ranged between 113 and 158 days, with an average age of 133.9 days (Table 1). Thus, all 14 fetuses were harvested during the 2nd trimester of pregnancy.

### 3.2. White-Tailed Deer Fetal Tissue

**Figure 1:**
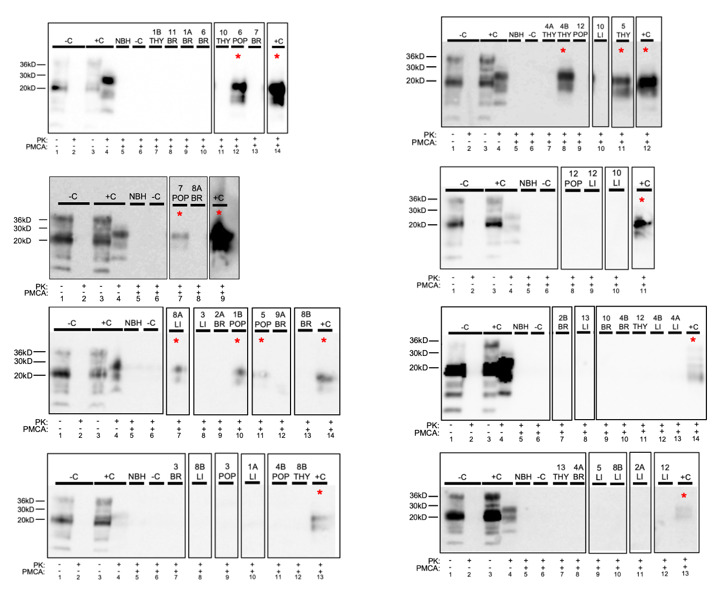
PrP^CWD^ detection in free-ranging white-tailed deer fetal tissues following seven rounds of sPMCA (method 1). Representative western blots for detection of PrP^CWD^ in liver (LI), popliteal lymph node (POP), and thymus (THY). sPMCA controls (10% homogenate, 7 rounds sPMCA) show complete proteinase K (PK) digestion of negative white-tailed deer brain homogenate (−C) and PK-resistant PrP^CWD^ in CWD+ brain homogenate (+C). Samples 1–9 were harvested from West Virginia; samples 10–13 were harvested from a non-endemic state, Georgia. Unamplified western blot assay controls show complete PK-digestion of CWD-negative white-tailed deer brain homogenate (−C; lane 2) and PK-resistant PrP^CWD^ in CWD+ white-tailed deer brain homogenate (+C; lane 4) (10% homogenate, undiluted, no sPMCA). PrP^CWD^ was not detected in brain (BR). * = sPMCA positive. NBH = normal brain homogenate. Sample type is identified along the top row of each western blot.

**Figure 2:**
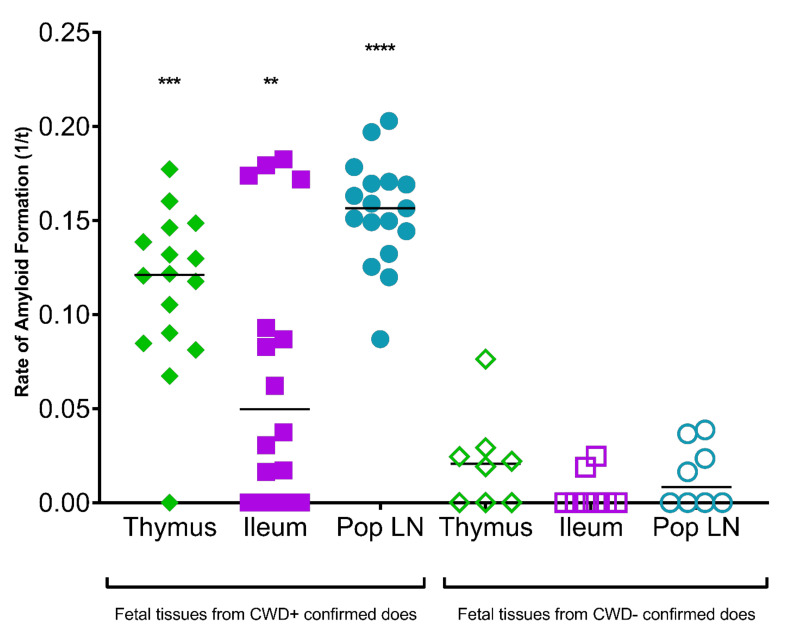
Detection of prion seeding activity by sPMCA/RT-QuIC (sPMCA method 2) in tissues harvested from fetuses of asymptomatic free-ranging CWD+ white-tailed deer does. Round 5 sPMCA reactions of fetal tissue samples (popliteal lymph node, ileum, and thymus) were analyzed by RT-QuIC. Statistically significant seeding activity was detected in all three fetal tissues analyzed. Asterisks indicate significance level (**** *p* < 0.0001, *** *p* < 0.001, ** *p* < 0.01).

Prion seeding activity was present in 7 of 14 (50%) fetuses harvested in the 2nd trimester of pregnancy in free-ranging naturally-infected CWD+ asymptomatic white-tailed deer (Table 1 and Table 2, Figure 1 and Figure 2). Prion seeding was detected in one of two fetal twins from does 1, 4, and 8, and neither twin was positive from does 2 and 9. (Table 2). Singleton fetal tissues from does 3, 5, 6, and 7 contained prion seeding activity. Fetal tissues containing prion seeding activity included liver, popliteal lymph node, ileum, and thymus (Figure 1 and Figure 2). Fetal tissue-type matched controls harvested from CWD negative does from a non-endemic region were free of PK-resistant PrP^CWD^ signal after sPMCA/WB and generated low to no sPMCA/RT-QuIC amyloid formation rates (Figure 1 and Figure 2). All fetal *Prnp* genotypes at codon 96 were GG.

**Table 1:** Demographic information from CWD+ asymptomatic, gravid white-tailed deer does and corresponding fetuses collected in Hampshire County, West Virginia to investigate potential for in utero transmission of prions. Four fetuses were harvested from four doe collected in Georgia, USA (fetuses 10–14) in the spring of 2003 for which no measurements were obtained.

Fetuses					Does					
Fetus ID	Sex	Weight (g)	Length (mm)	Gestation Length (Days)	Doe ID	Date Collected	Age (Years)	Weight (kg)	CWD IHC Result	
									RPLN	Obex
1A	M	790	289	127–129	1	March 2011	2.8	47	Pos	Pos
1B	F	727	283							
2A	M	820	289	119–129	2	March 2011	2.8	48	Pos	Pos
2B	F	510	257							
3	M	721	277	125	3	March 2011	3.8	46	Pos	Pos
4A	M	1115	332	139–143	4	April 2011	6.8	51	Pos	Pos
4B	F	948	318							
5	M	1579	374	157	5	April 2011	2.8	49	Pos	Pos
6	M	1647	378	158	6	April 2011	2.8	45	Pos	ND
7	M	1195	336	144	7	April 2011	1.8	41	Pos	Pos
8A	F	418	238	113–115	8	March 2012	3.8	55	Pos	Pos
8B	F	403	243							
9A	F	870	315	138	9	April 2012	2.8	40	Pos	Pos
9B	F	829	316	138						

**Table 2:** Prion detection in tissues harvested from fetuses of CWD+ white-tailed deer does. sPMCA reactions of fetal tissue samples (thymus, brain, ileum, popliteal lymph node, and liver) were analyzed by either western blot (sPMCA method 1; run 1; denoted by an asterisk (*)) or RT-QuIC (sPMCA method 2; runs 2 and 3). Positive reactions are highlighted in red. Brain homogenates were 20% *w/v* in PBS. Other tissue homogenates were 10% *w/v* in PBS with the following exceptions: thymus from 2A, 4A, and 5 (20%), popliteal LN from 9A and 9B (2.5% and 5%), and ileum from 1B and 9B (20%). Cells were left blank for the following reasons: tissues were not collected or tissues were depleted, preventing analysis.

Fetus ID	sPMCA Run #	Thymus	Brain	Ileum	Popliteal LN	Liver
1A	1 *		Neg			Neg
	2	Neg	Neg	Neg	Neg	
	3	Neg	Neg	Neg	Neg	
1B	1 *	Neg			**Pos**	
	2	Neg	Neg	Neg	Neg	
	3		Neg	Neg		
2A	1 *		Neg			Neg
	2	Neg	Neg	Neg	Neg	
	3	Neg		Neg	Neg	
2B	1 *		Neg			
	2	Neg	Neg	Neg	Neg	
	3	Neg		Neg	Neg	
3	1 *		Neg			Neg
	2		Neg	Neg	**Pos**	
	3		Neg	Neg	**Pos**	
4A	1 *	Neg	Neg			Neg
	2	Neg	Neg	Neg	Neg	
	3	Neg	Neg	Neg	Neg	
4B	1 *	**Pos**	Neg		Neg	Neg
	2		Neg	Neg	Neg	
	3			Neg		
5	1 *	**Pos**			**Pos**	Neg
	2	Neg	Neg	Neg	Neg	
	3	Neg	Neg	Neg		
6	1 *		Neg		**Pos**	
	2		Neg	**Pos**	Neg	
	3		Neg	Neg	Neg	
7	1 *		Neg		**Pos**	
	2	**Pos**	Neg	Neg	Neg	
	3	Neg		Neg	Neg	
8A	1 *		Neg			**Pos**
	2		Neg	**Pos**		
	3			Neg		
8B	1 *	Neg	Neg			Neg
	2	Neg	Neg	Neg		
	3	Neg	Neg	Neg		
9A	1 *		Neg			
	2	Neg		Neg		
	3	Neg	Neg	Neg		
9B	1 *					
	2	Neg		Neg	Neg	
	3	Neg		Neg	Neg	

## 4. Discussion

The continued geographical expansion of CWD in free-ranging cervid populations prompted us to investigate the role of maternally-derived infections in the facile transmission of this neurodegenerative prion disease. sPMCA is a highly sensitive and specific method of prion detection [30] that is superior to both IHC and western blot analysis [29]. Our previous studies, employing sPMCA, demonstrated the presence of CWD prions in fetal tissues harvested from experimentally infected muntjac [24,25] and naturally exposed free-ranging elk [26]. We have extended these works to determine the potential for mother-to-offspring transmission in naturally exposed free-ranging white-tailed deer. Here we demonstrate prebirth CWD exposure in white-tailed deer. Prion seeds were present in 50% of fetuses (7 of 14) collected from the uteri of 7 of 9 (78%) asymptomatic naturally exposed free-ranging CWD+ white-tailed does. These results are strikingly similar to our previous findings in free-ranging elk [26].

All of the fetuses in this study were opportunistically harvested during the second trimester of pregnancy. Prion seeds were found in an array of fetal tissues, suggesting broad distribution within the fetus during this gestational stage. Studies demonstrating PrP^Scrapie^ deposition in fetal tissues collected from scrapie-infected sheep provide further evidence for prion distribution within fetal tissues of prion-infected dams [18,30]. We and others have since demonstrated the presence of prions in early gestational in utero-derived fetal tissues of CWD-infected cervids [24,26,31]. CWD positive fetal tissues demonstrated by all three studies include thymus and liver. The current study in free-ranging WTD and our previous study in free-ranging elk [26] identified ileum and popliteal lymph node to contain CWD prion seeding capacity not demonstrated in farmed WTD [31]. No or limited tissue availability in this study prevented further analysis of several tissue types deemed CWD positive by previous work in naturally-exposed cervids (WTD and elk) including lung, brain, spleen and placenta, and maternally-derived tissues including uterus, umbilical cord, and cotyledon [26,31]. The studies above represent three cervid species; an experimental muntjac model, naturally-exposed farmed and free-ranging WTD, and free ranging elk. These findings further emphasize that offspring of three CWD-infected cervid species are exposed to prions long before exposure to contaminated environments or postpartum maternal secretions and excretions.

There are lingering questions about the biological relevance of these findings. The most compelling question is whether the presence of prion seeds in fetal tissues leads to CWD infection and disease progression in offspring born to CWD-infected cervids. In an attempt to estimate a sample’s infectious titer, we previously compared elk (E2) brain seventh round sPMCA product and E2 mouse bioassay data. We found that the extrapolated infectivity titer [0.32LD_50_ U (gram tissue) ^−1^] of a 10^−12^ dilution of E2 brain homogenate is the seeding dose for seven rounds of PMCA [26]. We further compared conventional and amplification CWD detection assays using a WTD brain pool (CBP6), demonstrating combined use of sPMCA plus RT-QuIC increased CWD detection by 2 logs [29]. The CWD starting materials differed between these studies, as well as incorporation of 5th vs. 7th round PMCA product as the starting seed for our RT-QuIC analysis in this study, making a direct comparison challenging. With this said, our experimental studies in muntjac demonstrated the presence of infectivity (mouse bioassay) within the pregnancy microenvironment of asymptomatic CWD-infected does [25]. We also revealed PrP^CWD^ deposition (IHC) within lymphoid biopsies of offspring born to asymptomatic CWD-infected does as early as 42 days post birth, with manifestation of clinical terminal CWD in 2–5 years [24]. Bioassay studies are underway to further assess maternal and fetal tissues from free-ranging WTD to provide additional insights.

Here we show evidence in three white-tailed does for prion accumulation in one of two fetuses in a twin set. Similar findings have been reported in fetal tissues harvested from scrapie-infected sheep [11]. This suggests the potential for a more focused infection within the pregnancy microenvironment, perhaps within specific placentomes that support fetal growth. The results of our experimental maternal infection studies in muntjac [25] and investigation of free-ranging elk [26] provide evidence that placentome prion seeding activity can vary within the same pregnancy, with some placentomes containing prions seeds, while other placentomes do not. Studies conducted in the scrapie system have targeted PrP^Scrapie^ deposition within the placentome structure [11] with PrP^Scrapie^ accumulation present within all regions of the placentome dependent upon the pregnancy stage. Cervid pregnancies are typically supported by 5 to 6 placentomes while sheep pregnancies are supported by 30 or more placentomes. The placentome structure permits the exchange of nutrients and waste between mother and fetus throughout pregnancy [32,33]. While the placental structure provides a barrier for the transfer of agents between mother and fetus, leaky or natural breaks within the placental structure are known to occur, creating small pools of blood within the maternal-fetal interface [11]. Blood is known to harbor prion infection [34,35,36,37,38]. Fetal-derived trophoblast cells are phagocytic and motile [39,40,41,42,43]. We hypothesize that fetal derived trophoblast cells enter blood-pooled spaces within the placentome, phagocytose prion carrying blood cells, and transport them back to the fetus.

Uterine prion infection would also be an obvious focus of maternal infection that may contribute to fetal infections. Previous findings employing our maternal infection muntjac model, where pregnancy timing and maternal/fetal prion deposition could be closely monitored, suggests that prion seeds are present within the placentome and fetus prior to the uterus [25]. These findings support the need for further investigation at the maternal-fetal interface seeking mechanisms of prion transfer from mother to offspring.

*Prnp* polymorphisms have been shown to play a role in both scrapie and CWD susceptibility [44,45,46]. The white-tailed deer fetuses in this study were all codon 96 GG, and thus from current understanding, carry a genotype that supports CWD infection. Further investigation of *Prnp* polymorphisms is ongoing to determine if additional genetic determinants may be contributing to our findings.

CWD is the most efficiently transmitted of the prion diseases [47]. The role of mother-to-offspring CWD transmission in free-ranging cervid populations remains largely unknown. Understanding the importance of this potential prion transmission route in free-ranging white-tailed deer populations is important to informing control strategies, as well as projecting CWD spread and potential impacts. The findings from this study in free-ranging white-tailed deer, coupled with our previous findings in free-ranging elk, provide the basis for continued exploration of the role vertical transmission may play in cervid populations across North America and other regions of the world, and may broaden our perspective of the transmission dynamics for all prion diseases.

## Data Availability

Data is contained within the article or available upon request from the corresponding author.

## References

[1] Mathiason C.K., Powers J.G., Dahmes S.J., Osborn D.A., Miller K.V., Warren R.J., Mason G.L., Hays S.A., Hayes-Klug J., Seelig D.M. (2006). Infectious prions in the saliva and blood of deer with chronic wasting disease. Science.

[2] Tamgüney G., Miller M.W., Wolfe L.L., Sirochman T.M., Glidden D., Palmer C., Lemus A., DeArmond S.J., Prusiner S.B. (2009). Asymptomatic deer excrete infectious prions in faeces. Nature.

[3] Safar J.G., Lessard P., Tamgüney G., Freyman Y., Deering C., Letessier F., DeArmond S.J., Prusiner S.B. (2008). Transmission and detection of prions in feces. J. Infect. Dis..

[4] Haley N.J., Seelig D.M., Zabel M.D., Telling G.C., Hoover E.A. (2009). Detection of CWD prions in urine and saliva of deer by transgenic mouse bioassay. PLoS ONE.

[5] Hoinville L.J., Tongue S.C., Wilesmith J.W. (2010). Evidence for maternal transmission of scrapie in naturally affected flocks. Prev. Vet. Med..

[6] Dickinson A.G., Young G.B., Stamp J.T., Renwick C.C. (1965). An analysis of natural scrapie in Suffolk sheep. Heredity.

[7] Dickinson A.G.S.J., Renwich C.C. (1974). Maternal and lateral transmission of scrapie in sheep. J. Comp. Pathol..

[8] Andreoletti O.L.C., Chabert A., Monnereau L., Tabouret G., Lantier F., Berthon P., Eychenne F., Lafond-Benestad S., Elsen J.M., Schelcher F. (2002). PrPSc accumulation in placentas of ewes exposed to natural scrapie: Influence of foetal PrP genotype and effect on ewe-to- lamb transmission. J. Gen. Virol..

[9] O’Rourke K.I., Zhuang D., Truscott T.C., Yan H., Schneider D.A. (2011). Sparse PrP(Sc) accumulation in the placentas of goats with naturally acquired scrapie. BMC Vet. Res..

[10] Tuo W., Zhuang D., Knowles D.P., Cheevers W.P., Sy M.S., O’Rourke K.I. (2001). Prp-c and Prp-Sc at the fetal-maternal interface. J. Biol. Chem..

[11] Tuo W., O’Rourke K.I., Zhuang D., Cheevers W.P., Spraker T.R., Knowles D.P. (2002). Pregnancy status and fetal prion genetics determine PrPSc accumulation in placentomes of scrapie-infected sheep. Proc. Natl. Acad. Sci. USA.

[12] Onodera T., Ikeda T., Muramatsu Y., Shinagawa M. (1993). Isolation of scrapie agent from the placenta of sheep with natural scrapie in Japan. Microbiol. Immunol..

[13] Lacroux C., Corbière F., Tabouret G., Lugan S., Costes P., Mathey J., Delmas J.M., Weisbecker J.L., Foucras G., Cassard H. (2007). Dynamics and genetics of PrPSc placental accumulation in sheep. J. Gen. Virol..

[14] Foster J.D., Goldmann W., Hunter N. (2013). Evidence in sheep for pre-natal transmission of scrapie to lambs from infected mothers. PLoS ONE.

[15] Alverson J., O’Rourke K.I., Baszler T.V. (2006). PrPSc accumulation in fetal cotyledons of scrapie-resistant lambs is influenced by fetus location in the uterus. J. Gen. Virol..

[16] Race R., Jenny A., Sutton D. (1998). Scrapie infectivity and proteinase K-resistant prion protein in sheep placenta, brain, spleen, and lymph node: Implications for transmission and antemortem diagnosis. J. Infect. Dis..

[17] Schneider D.A., Madsen-Bouterse S.A., Zhuang D., Truscott T.C., Dassanayake R.P., O’Rourke K.I. (2015). The placenta shed from goats with classical scrapie is infectious to goat kids and lambs. J. Gen. Virol..

[18] Spiropoulos J., Hawkins S.A., Simmons M.M., Bellworthy S.J. (2014). Evidence of in utero transmission of classical scrapie in sheep. J. Virol..

[19] Maddison B.C., Baker C.A., Rees H.C., Terry L.A., Thorne L., Bellworthy S.J., Whitelam G.C., Gough K.C. (2009). Prions are secreted in milk from clinically normal scrapie-exposed sheep. J. Virol..

[20] Konold T., Moore S.J., Bellworthy S.J., Simmons H.A. (2008). Evidence of scrapie transmission via milk. BMC Vet. Res..

[21] Konold T., Moore S.J., Bellworthy S.J., Terry L., Thorne L., Ramsay A., Salguero F.J., Simmons M.M., Simmons H. (2013). Evidence of effective scrapie transmission via colostrum and milk in sheep. BMC Vet. Res..

[22] Madsen-Bouterse S.A., Highland M.A., Dassanayake R.P., Zhuang D., Schneider D.A. (2018). Low-volume goat milk transmission of classical scrapie to lambs and goat kids. PLoS ONE.

[23] Ligios C., Cancedda M.G., Carta A., Santucciu C., Maestrale C., Demontis F., Saba M., Patta C., DeMartini J.C., Aguzzi A. (2011). Sheep with scrapie and mastitis transmit infectious prions through the milk. J. Virol..

[24] Nalls A.V., McNulty E., Powers J., Seelig D.M., Hoover C., Haley N.J., Hayes-Klug J., Anderson K., Stewart P., Goldmann W. (2013). Mother to offspring transmission of chronic wasting disease in reeves’ muntjac deer. PLoS ONE.

[25] Nalls A.V., McNulty E., Hoover C.E., Pulscher L.A., Hoover E.A., Mathiason C.K. (2017). Infectious Prions in the Pregnancy Microenvironment of CWD-infected Reeves’ Muntjac Deer. J. Virol..

[26] Selariu A., Powers J.G., Nalls A., Brandhuber M., Mayfield A., Fullaway S., Wyckoff C.A., Goldmann W., Zabel M.M., Wild M.A. (2015). In utero transmission and tissue distribution of chronic wasting disease-associated prions in free-ranging Rocky Mountain elk. J. Gen. Virol..

[27] Stewart P., Campbell L., Skogtvedt S., Griffin K.A., Arnemo J.M., Tryland M., Girling S., Miller M.W., Tranulis M.A., Goldmann W. (2012). Genetic predictions of prion disease susceptibility in carnivore species based on variability of the prion gene coding region. PLoS ONE.

[28] Hamilton R.J., Tobin M.L., Moore W.G. Aging fetal white-tailed deer. Proceedings of the Annual Conference Southeastern Association of Fish and Wildlife Agencies.

[29] McNulty E., Nalls A.V., Mellentine S., Hughes E., Pulscher L., Hoover E.A., Mathiason C.K. (2019). Comparison of conventional, amplification and bio-assay detection methods for a chronic wasting disease inoculum pool. PLoS ONE.

[30] Saa P., Castilla J., Soto C. (2006). Presymptomatic detection of prions in blood. Science.

[31] Bravo-Risi F., Soto P., Eckland T., Dittmar R., Ramírez S., Catumbela C.S.G., Soto C., Lockwood M., Nichols T., Morales R. (2021). Detection of CWD prions in naturally infected white-tailed deer fetuses and gestational tissues by PMCA. Sci. Rep..

[32] Garza M.C., Fernández-Borges N., Boles R., Badiola J.J., Castilla J., Monleon E. (2011). Detection of PrPres in Genetically Susceptible Fetuses from Sheep with Natural Scrapie. PLoS ONE.

[33] Slack J.M.W. (2013). Essential Developmental Biology.

[34] Hunter N., Foster J., Chong A., McCutcheon S., Parnham D., Eaton S., MacKenzie C., Houston F. (2002). Transmission of prion diseases by blood transfusion. J. Gen. Virol..

[35] Houston F., Foster J.D., Chong A., Hunter N., Bostock C.J. (2000). Transmission of BSE by blood transfusion in sheep. Lancet.

[36] Mathiason C.K., Powers J.G., Dahmes S.J. (2006). Don’t kiss that deer. Compend. Contin. Educ. For. Pract. Vet..

[37] Mathiason C.K., Hayes-Klug J., Hays S.A., Powers J., Osborn D.A., Dahmes S.J., Miller K.V., Warren R.J., Mason G.L., Telling G.C. (2010). B Cells and Platelets Harbor Prion Infectivity in the Blood of Deer Infected with Chronic Wasting Disease. J. Virol..

[38] Peden A.H., Head M.W., Ritchie D.L., Bell J.E., Ironside J.W. (2004). Preclinical vCJD after blood transfusion in a PRNP codon 129 heterozygous patient. Lancet.

[39] Myagkaya G., Schellens J.P. (1981). Final stages of erythrophagocytosis in the sheep placenta. Cell Tissue Res..

[40] Wooding F.B. (1982). The role of the binucleate cell in ruminant placental structure. J. Reprod. Fertil. Suppl..

[41] Wooding F.B. (1992). Current topic: The synepitheliochorial placenta of ruminants; binculeate cell fusions and hormone production. Placenta.

[42] Wooding F.B. (1994). Marshall’s Physiology of Reproduction.

[43] Wooding F.B., Morgan G., Adam C.L. (1997). Structure and function in the rumiant synepithelialchorial placenta: Central role of the trophoblast binucleate cll in deer. Microsc. Res. Tech..

[44] Soto P., Claflin I.A., Bursott A.L., Schwab-McCoy A.D., Bartz J.C. (2021). Cellular prion protein gene polymorphisms linked to differential scrapie susceptibility correlate with distinct residue connectivity between secondary structure elements. J. Biomol. Struct. Dyn..

[45] Hagenaars T.J., Melchior M.B., Bossers A., Davidse A., Engel B., van Zijderveld F.G. (2010). Scrapie prevalence in sheep of susceptible genotype is declining in a population subject to breeding for resistance. BMC Vet. Res..

[46] Haley N., Donner R., Merrett K., Miller M., Senior K. (2021). Selective Breeding for Disease-Resistant PRNP Variants to Manage Chronic Wasting Disease in Farmed Whitetail Deer. Genes.

[47] Benestad S.L., Telling G.C. (2018). Chronic wasting disease: An evolving prion disease of cervids. Handb. Clin. Neurol..

